# Profile of male acute admissions at a state psychiatric hospital in the Western Cape, South Africa

**DOI:** 10.4102/sajpsychiatry.v32i0.2710

**Published:** 2026-08-31

**Authors:** Anthea Payne Maziena, Muneeb Salie, Liezl Koen, Dana Niehaus, Petrus Steyn

**Affiliations:** 1 Department of Psychiatry, Faculty of Medicine and Health Sciences, Stellenbosch University, Cape Town, South Africa; 2 Panorama Healthcare Psychiatry, Cape Town, South Africa

**Keywords:** inpatient, psychiatric services, readmissions, male acute unit, length of stay, crisis discharge

## Abstract

**Background:**

Inpatient psychiatric services in South Africa are faced with challenges such as high demand for beds, rapid discharges, limited community services upon discharge and high readmission rates.

**Aim:**

To provide a profile of the demographic and clinical characteristics of the mental health users of inpatient male acute psychiatric services.

**Setting:**

The male acute unit at Stikland Hospital.

**Methods:**

A retrospective folder review of all patients discharged (*n* = 693) from the male acute psychiatric unit at Stikland Hospital between 01 February 2022 and 31 January 2023, and followed up for 1 year post-discharge.

**Results:**

Most of the admissions were young males (29 [23–35] years), single (89%) and unemployed (80.8%), with no secondary-level education (65.4%). Thirty per cent of admissions were admitted directly to the unit, and most patients presented with a diagnosis of schizophrenia (42.1%). Almost half (47.8%) of all admissions were patients previously admitted to Stikland Hospital, and more than one quarter (27.6%) were readmitted within 1 year following discharge. Most admissions had a short length of stay (36 [27–53] days) and were crisis-discharged (63.5%). The use of long-acting injectables (LAI) was highly prevalent (73.4%).

**Conclusion:**

Current inpatient psychiatric services are functioning as crisis intervention units. The study provides a foundation for developing context-specific, tailored strategies to be integrated into the discharge planning processes within the existing healthcare system.

**Contribution:**

Current system-related factors, such as bed shortages, lead to short length-of-stays (LOS) and crisis discharges, which likely contribute to a higher rate of readmissions.

## Introduction

Despite widespread deinstitutionalisation, the demand for inpatient psychiatric care still far outweighs supply, particularly for patients who pose a significant risk, refuse necessary treatment or have severe symptoms impairing daily functioning.^1,2,3,4^ In South Africa, deinstitutionalisation following the advent of democracy in the mid-1990s resulted in a substantial reduction in inpatient beds, with chronic psychiatric beds in Gauteng halving between 1994 and 2004.^5^ This reduction in inpatient capacity has led to high bed occupancy, shortages and shorter hospital stays.^1,2,6,7^ Admission practices have been influenced, with inpatient units increasingly admitting patients with severe psychopathology, particularly psychotic and substance use disorders, while fewer patients with non-psychotic presentations are admitted.^8,9^ Even in settings with relatively good community support, bed shortages contribute to declining patient well-being in the community, longer lengths of stay once admitted, and pressure to discharge patients who remain symptomatic.^6,10,11^ As a result, only the most severely ill patients are admitted,^9^ and they are often discharged rapidly, transforming inpatient units into short-term crisis-intervention services.^12,13^

Ongoing shortages of adequate community mental health resources, particularly in rural areas, remain a major constraint in South Africa.^14,15^ Consequently, despite policy aims to move away from a hospital-centred model, inpatient psychiatric care remains a critical component in South Africa and other low- to middle-income countries (LMICs), highlighting the importance of efficient use of inpatient services in resource-limited settings.^1,14,16^ Some international organisations have set the maximum bed occupancy in a psychiatric unit at 85% to ensure patient and staff safety.^10^ However, psychiatric hospitals in the Western Cape typically operate at 100% or higher bed occupancy, according to the Mental Health Dashboard on the Single Patient Viewer platform.^17^

Inpatient clinical teams may be required to periodically review and adjust their practices in response to ongoing bed-capacity constraints within the South African state psychiatric sector. Stikland Hospital follows a ‘crisis discharge policy’, adopted by the associated psychiatric hospital management team in the Western Cape province, South Africa, which governs the practice of premature discharge, as standard operating procedure since 2003.^18^ A ‘crisis discharge’ is made when no inpatient beds are available, but a patient requires urgent admission. Some safeguards exist to limit risk with crisis discharges, including that the patient should be the most stable in the ward, not pose any immediate risks to him-/herself or others, be less unwell than the patient requiring admission and be able to follow up with practical plans in place.

Given the above, it is not surprising that readmission rates for psychiatric patients remain high in South Africa. Almost one in four patients is readmitted within 3 months of psychiatric discharge, with readmission costing an estimated 1.5 billion rand in 2016/2017, equal to almost a fifth of the mental healthcare budget.^14^ Crisis discharges and similar practices have the potential to contribute to relapse, which disrupts psychosocial rehabilitation, employment and other psychosocial support, and increases distress for the patient and family, risk for treatment resistance, and costs of care.^19,20,21,22^ Demographic and clinical factors that have been associated with a higher risk of relapse include male gender, a schizophrenia diagnosis, a history of an admission for stimulant use disorders before the index admission with psychosis, psychiatric comorbidity, treatment adherence, previous hospitalisation and lack of housing.^23,24,25^ There are also system-related factors associated with relapse, which include limitations of available inpatient care programmes, lack of availability of prescribed medication in the community, poor post-discharge care, lack of comprehensive community care, lack of coordination with community mental health workers, disruption to social security benefits and locations of the hospitals and patients.^11,24,25^ Readmission is commonly used as a measurement of relapse.^26,27^ Readmission can be conceptualised as the outcome of an individual’s specific needs exceeding the capacity of the environment and support system.^7^ Early readmission is therefore often seen as an indication of too early discharge, poor quality of psychiatric care or lack of discharge coordination.^14,28^

The Western Cape province has the highest documented lifetime prevalence of common mental disorders in South Africa. Despite this, the Western Cape has only 0.89 psychiatrists per 100 000 of the uninsured population compared to a global average of 3.96 per 100 000 of the population.^14^ The majority of South Africans with serious mental illness (SMI) receive little to no psychiatric care. In settings with limited community resources, this places an additional responsibility on inpatient services to stabilise patients adequately.^29^

Stikland Hospital is one of three specialised state psychiatric hospitals in the Western Cape province of South Africa. The Tygerberg South substructure falls within the service area of this specialised facility. However, it currently lacks a dedicated district-level psychiatric admission facility, resulting in this specialised hospital also providing services that are typically delivered at the district-level for this substructure. Consequently, some patients are admitted directly from the community to specialised-level care, a process commonly referred to as a ‘direct admission’, in other words, without a preceding admission at a district hospital. District-level facilities would ordinarily assist with the initial stabilisation of medical comorbidities and the early management and containment of acute psychiatric presentations before transfer to specialised services. Direct admissions likely contribute to the emergency management demands placed on tertiary acute psychiatric units and could add to the workload of clinical staff in these settings.

Understanding the current challenges facing this mental health system is therefore important not only within our own context but also in the broader public mental health service in South Africa and similar LMICs in general. Challenges such as high bed occupancy, high demand for beds, and limited community resources place conflicting pressures on acute services, leading to rapid discharges and, in our setting, an official crisis discharge policy. A description of this cohort and an evaluation of readmission rates within this service may provide important insights to inform current clinical practices and guide the development of strategies aimed at reducing readmissions.

## Research methods and design

### Study design

A retrospective folder review was conducted of all patients discharged from the male acute psychiatric unit at Stikland Hospital between 01 February 2022 and 31 January 2023, with 1-year post-discharge follow-up. This study period was decided as it was 1 year before the implementation of a change in the ward programme. The SMART (Stikland Male Acute Remodelling Trial) programme was started in February 2023 in an attempt to improve the quality of services delivered, limit readmissions and promote recovery and retention in care. The programme included an increase in the amount of therapeutic interventions offered in the male acute service, as well as more active attempts to trace patients who had been lost to follow-up services.

### Study setting

Stikland Hospital is a state psychiatric hospital situated in Bellville, Western Cape province, South Africa.^30^ The facility provides all levels of psychiatric care to a part of the Tygerberg East metro region, as well as the Winelands rural region. Even though the male acute service only accounts for 20.5% of all beds at Stikland Hospital, the service, on average, accounts for more than 43% of all admissions, as well as 69% of all acute admissions, within the context of consisting of 59.5% of all acute funded beds.

### Data collection

Data were extracted from patient folders and the Electronic Continuation of Care Record (eCCR) and Single Patient Viewer (SPV), two applications of the Western Cape Department of Health and Wellness.

### Data points

The first time a patient was discharged during the study period was assigned as the ‘index discharge’ and the admission related to it as the ‘index admission’. All following admissions to either Stikland Hospital or a district hospital psychiatric ward were assigned as ‘subsequent admissions’ and each numbered according to its chronological relationship to the others. The total duration of each subsequent admission in days was recorded. All patients were followed up for 1 year after the date of the index discharge to record follow-up and subsequent admissions.

### Data analysis

Data were analysed using IBM SPSS Statistics, Version 28 ([computer program]. Armonk, NY: IBM Corporation; 2021). Continuous variables were summarised as mean and standard deviation or median and interquartile ranges (IQR), while categorical variables were summarised as counts and percentages. As this was a descriptive study, no inferential statistical analyses were conducted.

### Ethical considerations

Ethical approval was obtained from the Stellenbosch University Health Research Ethics Committee (Reference number: N23/11/134). Approval to access relevant medical records at Stikland Hospital was obtained from the Provincial Health Research Committee and management of Stikland Hospital (Reference number: WC_202311_023). All endeavours to ensure the safety and confidentiality of patient records were followed, and all collected data were anonymised by the removal of direct identifiers. A waiver of informed consent was granted, given the retrospective nature of the study. This study was conducted in accordance with the Declaration of Helsinki^31^ and the South African Good Clinical Practice Guidelines.^32^

## Results

A total of 712 discharges were identified from hospital records. Of these, 10 discharges were transferred to a different hospital for further tertiary care, six discharges were found to have incomplete records, two discharges had duplicate records, and one discharge died during admission. A total of 693 discharges were analysed. The median (IQR) age of the sample was 29 (23–35) years (range 18–59). Most of the participants were not in a relationship (*n* = 617, 89%) and lived with family (*n* = 602, 86.9) (Table 1). Just over two-thirds of participants did not complete schooling (*n* = 453, 65.4%), most were unemployed (*n* = 650, 80.8%) and only 23.2% (*n* = 161) were recipients of a disability grant.

**TABLE 1:** Demographic characteristics of patients (*N* = 693) admitted to the male acute service at Stikland Hospital from 01 February 2022 to 31 January 2023.

Variable	*n*	%
**Relationship status**	672	-
Not in a relationship†	617	89.0
In a relationship‡	55	7.9
**Highest level of education**	617	-
Did not complete schooling§	453	65.4
Completed high school	131	18.9
Vocational training or tertiary qualification	33	4.8
**Employment status**	653	-
Unemployed	650	80.8
Employed¶	93	13.4
**Disability grant**	431	-
No	270	39.0
Yes	161	23.2
**Housing and support**	640	-
Homeless	7	1.0
Lives alone	17	2.5
Lives with family	602	86.9
Lives with someone else who is not family	14	2.0

† , Composite of ‘single’, ‘divorced’, ‘widowed’ and separated.

‡ , Composite of ‘married/cohabitating’ and ‘other relationship’.

§ , Composite of ‘no formal schooling’, ‘primary school’ and ‘secondary school’.

¶ , Composite of ‘formally’ and ‘informally’ employed.

Almost half (*n* = 331, 47.8%) of all index admissions were previously admitted to Stikland Hospital (Table 2), with patients having a median (IQR) of 3 (1–5) previous admissions. The duration of previous Stikland Hospital admissions ranged between 39 days and 45 days, and the median (IQR) total length of stay for previous Stikland Hospital admissions was 123 (56–224) days. More than two-thirds (*n* = 480, 69.3%) of the patients were referred from other hospitals to Stikland Hospital for their index admission. The median (IQR) length of stay of the index Stikland Hospital admission was 36 (27–53) days. A total of 30.7% (*n* = 213) of all male acute admissions were admitted directly into the service without being admitted for a period of time in a referral centre.

**TABLE 2:** Number and duration of admissions: pre-index, index and post-index (*N* = 693).

Variable	*n*	%	Median	IQR	Range
**Pre-index**					
Previously admitted to Stikland Hospital (STH), yes	331	47.8	-	-	-
Number of previous Stikland Hospital admissions	-	-	3	1–5	1–5
Length of stay of the 1st previous Stikland Hospital admission, days	331	-	45	29–74	1–449
Length of stay of the 2nd previous Stikland Hospital admission, days	234	-	44	28–70	0–366
Length of stay of the 3rd previous Stikland Hospital admission, days	170	-	42	28–72	0–338
Length of stay of the 4th previous Stikland Hospital admission, days	143	-	44	29–65	1–332
Length of stay of the 5th previous Stikland Hospital admission, days	121	-	39	29–56	1–195
Total length of stay of previous Stikland Hospital admissions, days	331	-	123	56–224	2–924
**Index**					
Length of stay of referring hospital admission, days	478†	-	17	12–23	1–205
Length of stay of Index Stikland Hospital admission, days	693	-	36	27–53	2–299
Total Length of stay of hospital admission, days	693	-	50	38–69	2–299
**Post-index**					
**Duration between Stikland Hospital index discharge and Stikland Hospital readmission, days**	191	-	137	68–238	3–362
Length of stay of the 1st Stikland Hospital readmission, days	191	-	43	28–60	0–378
Length of stay of the 2nd Stikland Hospital readmission, days	138	-	39	24–61	0–554
Length of stay of the 3rd Stikland Hospital readmission, days	77	-	40	27–66	0–331
Length of stay of the 4th Stikland Hospital readmission, days	40	-	37	25–50	0–244
**Duration between Stikland Hospital index discharge and psychiatric district hospital readmission, days**	158	-	180	66–244	2–364
Length of stay of the 1st psychiatric district hospital readmission, days	158	-	16	11–24	1–73
Length of stay of the 2nd psychiatric district hospital readmission, days	49	-	15	8–21	0–83
Length of stay of the 3rd psychiatric district hospital readmission, days	24	-	18	8–28	1–75
Length of stay of the 4th psychiatric district hospital readmission, days	6	-	8	5–24	4–39
Length of stay of the 5th psychiatric district hospital readmission, days	2	-	12	1–23	1–23
**Duration between Stikland Hospital index discharge and other psychiatric hospital readmission, days**	8	-	197	164–257	120–479
Length of stay of the 1st readmission to a psychiatric hospital (other than Stikland Hospital) after index discharge, days	8	-	29	19–54	2–68
Length of stay of the 2nd readmission to a psychiatric hospital (other than Stikland Hospital) after index discharge, days	1	-	21	-	-

IQR, interquartile range.

† , Admission date at the referring hospital was missing for two patients.

For readmission to Stikland Hospital within 1 year after their index admission, 191 patients had at least one post-index readmission, and 40 patients had four post-index readmissions (Table 2). The median (IQR) time to first readmission was 137 (68–238) days. The median length of stay of post-index readmissions to Stikland Hospital ranged between 37 and 43 days. For readmission to a psychiatric unit at a district hospital, 158 patients had at least one post-index readmission, and two patients had five post-index readmissions. The median (IQR) time to first readmission was 180 (66–244) days. The median length of stay of post-index readmissions to a district hospital ranged between 8 days and 18 days. Eight patients were readmitted to another psychiatric hospital (other than Stikland Hospital) for a median (IQR) length of stay of 29 (19–54) days after their index admission.

The most common primary psychiatric diagnoses at Stikland Hospital index discharge were schizophrenia (*n* = 292, 42.1%), substance-induced psychotic disorder (*n* = 135, 19.5%) and schizophreniform disorder (*n* = 87, 12.6%) (Table 3). An ‘other’ (*n* = 162, 23.4%) and substance use disorder (*n* = 134, 19.3%) were the most common secondary psychiatric diagnoses. Just over one-fifth of participants (*n* = 159, 22.9%) presented with a first episode of psychosis. Of these patients with a first episode of psychosis, 30 (18.9%) were readmitted to a psychiatric hospital or unit within 1 year post-index discharge. Forty per cent of participants (*n* = 280) had untreated psychosis for a median (IQR) of 30 (7–90) days. Almost two-thirds of participants were crisis-discharged (*n* = 440, 63.5%).

**TABLE 3:** Psychiatric diagnosis characteristics during the index admission/discharge (*N* = 693).

Psychiatric diagnosis	Primary		Secondary	

	*n*	%	*n*	%
Schizophrenia	292	42.1	-	-
Schizophreniform	87	12.6	-	-
Substance-induced psychotic disorder	135	19.5	2	0.3
Schizoaffective disorder	71	1.4	-	-
Psychosis due to another medical condition	10	1.4	-	-
Other psychotic disorders	14	2.0	-	-
Bipolar I disorder	61	8.8	-	-
Depressive disorder, excluding substance-induced	2	0.3	8	1.2
Adjustment disorder with depressed mood	-	-	3	0.4
Anxiety disorder	-	-	2	0.3
Obsessive-compulsive disorder	-	-	2	0.3
Substance use disorder	12	1.7	134	19.3
Major neurocognitive disorder	2	0.3	5	0.7
Personality disorder	1	0.1	11	1.6
Intellectual disability	1	0.1	9	1.3
Other	5	0.7	162	23.4
**Psychiatric characteristics**	-	-	**Median**	**IQR**
First episode psychosis at index admission	159	22.9	-	-
Readmitted within 1-year after index discharge, yes	30	18.9	-	-
Untreated psychosis, yes	280	40.4	-	-
Duration of untreated psychosis, days	-	-	30	7–90
Crisis discharged, yes	440	63.5	-	-

IQR, interquartile range.

Co-morbid medical conditions were infrequent, with hypertension (*n* = 52, 7.5%), COVID-19 (*n* = 50, 7.2%), pulmonary TB (*n* = 41, 5.9%) and syphilis (*n* = 34, 5.0%) being the most frequently observed (Table 4). A diagnosis of human immunodeficiency virus/acquired immunodeficiency syndrome (HIV/AIDS) was documented in 3.5% of admissions.

**TABLE 4:** Distribution of medical comorbidities among male acute admissions presenting with psychiatric disorders (*N* = 693).

Comorbidity	*n*	%
Hypertension	52	7.5
HIV/AIDS	25	3.5
*HIV^+^, not on ARVs*	14	2.0
*HIV^+^, on ARVs*	10	1.4
*HIV stage 1–3, including pulmonary TB*	1	0.1
Pulmonary TB	41	5.9
COVID-19	50	7.2
Syphilis	34	5.0
*Primary*	20	2.9
*Latent*	8	1.2
*Neurosyphilis*	6	0.9
Other	117	16.9

HIV, human immunodeficiency virus; AIDS, acquired immunodeficiency syndrome; ARV, antiretroviral; TB, tuberculosis; COVID-19, coronavirus disease 2019.

A high prevalence of substance use was noted in this population, with cannabis, methamphetamine and methaqualone being the substances most frequently used (Table 5). The prevalence of lifetime use of cannabis was 81.5% (*n* = 565), methamphetamines was 54.4% (*n* = 377) and methaqualone was 47.5% (*n* = 329). The prevalence of prior-month use of cannabis was 29.6% (*n* = 205), methamphetamines was 20.8% (*n* = 144) and methaqualone was 29.6% (*n* = 205).

**TABLE 5:** The distribution of substance use patterns among admissions presenting to a tertiary male acute psychiatric unit (*N* = 693).

Substances	Lifetime use		Prior-month use	

	*n*	%	*n*	%
Cannabis	565	81.5	205	29.6
Methamphetamines	377	54.4	144	20.8
Stimulants	62	8.9	46	6.6
Methaqualone	329	47.5	135	19.5
Alcohol	224	32.3	26	3.8
Nicotine	196	28.3	20	2.9
Other	22	3.2	7	1.0

Almost all patients were prescribed antipsychotics during their index admission/discharge (*n* = 688, 99.3%), while only 7.2% (*n* = 50) were prescribed an antidepressant (Table 6). Of those prescribed an antipsychotic, the median (IQR) number of antipsychotics was 2 (2–2), 73.4% (*n* = 505) were prescribed a long-acting injectable (LAI) and 22.7% (*n* = 156) were prescribed clozapine.

**TABLE 6:** Psychotropic medications prescribed (*N* = 693).

Type of psychotropic prescribed	Median	IQR	Min	Max	*n*	%
Antidepressants†	-	-	-	-	50	7.2
Number of prescribed antidepressants	1	1–1	1	2	-	-
Mood stabilisers‡	-	-	-	-	300	43.3
Number of prescribed mood stabilisers	1	1–1	1	3	-	-
Antipsychotics§	-	-	-	-	688	99.3
Number of prescribed antipsychotics	2	2–2	1	4	-	-
Prescribed a long-acting injectable¶ antipsychotic	-	-	-	-	505	73.4
Prescribed clozapine as an antipsychotic	-	-	-	-	156	22.7

IQR, interquartile range; Min., minimum; Max., maximum.

† , Includes: fluoxetine, citalopram, venlafaxine, mirtazapine and amitriptyline.

‡ , Includes: lithium, sodium valproate, carbamazepine and lamotrigine.

§ , Includes: haloperidol, chlorpromazine, flupenthixol depot, zuclopenthixol depot, amisulpride, risperidone, risperdal consta, olanzapine, quetiapine, aripiprazole and clozapine.

¶ , Includes: flupenthixol depot, zuclopenthixol depot and risperdal consta.

## Discussion

Stikland Hospital is a specialised psychiatric hospital in Bellville, Western Cape province, South Africa. The male acute service is the most highly pressured unit within the hospital, and this study aimed to provide a descriptive summary of the demographic and clinical characteristics of patients utilising this service. Most of the admissions were young, single and unemployed with no secondary-level education. Almost a third of all admissions were admitted directly into specialised care, and most patients presented with a diagnosis of schizophrenia. Most admissions had a short length of stay, were discharged as crisis discharges and were admitted as substance users. While the use of LAI was highly prevalent, readmissions remained a significant challenge within the service.

The index admissions had a median length of stay of 36 days, which is similar to the UK (35.9 days).^10^ A shorter length-of-stays (LOS) has been associated with substance abuse and psychiatrists managing high-volume caseloads, which are both significant factors in this population.^33^ Average LOS in most industrialised countries varies from 6.4–13 days in the US to about 35.9 days in the United Kingdom, compared to 54.4–79.7 days in Japan.^10,12,34^ The lack of post-discharge community coordination and transitional care in the Japanese system leads to a greater emphasis on full stabilisation of the patient’s condition during the inpatient admission and subsequently longer stays.^12^ The situation with regard to the lack of community resources is similar in many developing nations. In China, for example, psychiatric beds are limited and unequally distributed, which has been linked to long psychiatric admissions, with an average LOS of 50–60 days.^16^ A longer LOS is associated with the availability of beds, which is a significant limiting factor in our population. Stikland Hospital’s male acute unit always runs above 100% bed capacity with a long waiting list for admission. The continuous bed pressure leads to crisis discharges and, therefore, a shorter LOS. Considering the lack of community resources, a longer LOS would ideally assist with greater stabilisation before discharge; however, system-related factors such as bed pressure render a longer LOS unlikely.

Almost half of all admissions in this study were patients previously admitted to Stikland Hospital, and more than one quarter were readmitted within 1 year following discharge. A readmission rate of less than 15% within 28 days was previously suggested as an acceptable standard.^1^ Although relapse is expected as a part of the natural course of psychotic illness,^16,20^ almost a third of patients are readmitted into acute psychiatric services within 3 months of their discharge.^35^ In the context of increasing pressure on acute psychiatric services, readmission rates represent a critical area for targeted intervention, with efforts to reduce readmissions offering a potential means of alleviating strain on healthcare systems and improving continuity of care.

Niehaus et al.^18^ also reported results from a Stikland Hospital study in 2008, which showed that patients discharged as usual had a far lower risk of readmission compared to those discharged as ‘crisis discharges’. Despite this finding, the crisis discharge policy remains practised because of ongoing bed shortages for acute inpatient admissions. Our study showed that 63.5% of discharges within our study period were documented as crisis discharges. This remains a significant factor to consider when assessing the risk of readmission.

Schizophrenia was the most predominant diagnosis listed on discharge in this cohort. Zhan et al.^36^ analysed the results of the Global Burden of Disease study for schizophrenia, looking at trends from 1990 to 2021 and projections to 2050. The results found that between 1990 and 2021, the prevalence of schizophrenia increased from 13.62 million to 23.18 million, which reflects a 70% increase. The incidence of schizophrenia showed a 38.5% increase as it escalated from 883 000 to 1.223 million. The authors highlight the anticipated increase in the burden of schizophrenia as projections indicate a continuous rise through 2050. Policymakers are advised to revise policies and implement measures, which include the development of comprehensive systems to proactively address the anticipated increase.

Our study also showed that just over a fifth of the study population presented with a first episode of psychosis. Within the first episode psychosis group, 18.9% were readmitted within 1 year after discharge. The importance of early intervention in first episode psychosis remains an extensively studied topic, and it is well known that early intervention leads to improved prognosis. The review by Nikolai et al.^37^ highlighted the importance of reducing the duration of untreated psychosis and providing specialised early intervention treatment in first episode psychosis.

Non-psychiatric medical comorbidity is an interesting and seemingly important marker of poor mental health, and possibly acts as a precipitant to acute psychiatric episodes.^38^ Three of the four most frequently noted medical comorbidities in this study were infectious diseases. People with schizophrenia are known to be at an increased risk of infectious diseases.^39,40^ Anecdotal evidence from South Africa suggests that the stress of the COVID-19 pandemic was linked to an increase in medication discontinuation and schizophrenia relapses.^39^ In the initial COVID-19 post-pandemic period, there were increases noted in psychiatric admissions for psychosis.^41,42^ It seems likely that psychotic disorders continue to predominate psychiatric admissions in the post-COVID-19 era, leading to higher numbers of involuntary admissions and greater pressure on psychiatric hospitals.^41^

The South African Stress and Health (SASH) study, a large population-based mental health epidemiological survey, showed that the Western Cape had a 20.6% lifetime prevalence of substance use, the highest compared to other provinces within South Africa.^43^ Our study showed that substance use remains a highly prevalent condition within this population. Multiple substance use was previously associated with more frequent readmissions and a shorter LOS in schizophrenia spectrum disorders.^44^ Florentin et al.^45^ found that dual diagnosis (severe mental illness with a substance use disorder) patients had a significantly younger mean age at first hospitalisation and shorter average LOS per hospitalisation, but a greater total number of hospitalisations and hospital days when compared to non-substance users. Substance use interventions should be considered a priority in this service or post-discharge community planning, as they could be a contributing factor to readmission and worsening prognosis.

The use of antipsychotic polypharmacy has been shown to increase the risk for readmission when compared to antipsychotic monotherapy.^46^ In this cohort, the majority of patients were discharged on a LAI antipsychotic, and the median number of prescribed antipsychotics was two. Patel et al.^47^ showed that LAI antipsychotics used in psychotic disorders decreased readmission rates at 90 days and 1 year post-discharge at a state psychiatric hospital in the US. Only one-fifth of the cohort was prescribed clozapine, which is lower than the one-third of patients found to be on clozapine treatment in a tertiary hospital in Johannesburg, South Africa.^48^ A retrospective cohort study conducted in Taiwan between 2006 and 2021 showed that patients on a clozapine and an LAI combination had a longer time to rehospitalisation when compared to clozapine monotherapy or clozapine with other antipsychotic regimens.^49^ Further studies are required to compare the categories of medication use to readmission in this population.

A limitation of the study is the fact that conducting a retrospective study relies on the records kept by others. The accuracy of the data is dependent on several self-reported pieces of information and collateral information provided by family members. Retrospective data also limits inference of causality. Further studies are needed to provide deeper insight into factors associated with low-frequency and high-frequency service users.

## Conclusion

Inpatient psychiatric care remains an essential service in South Africa, but current services are functioning as crisis intervention units. Most admissions are as a result of psychotic disorders, and substance use remains highly prevalent in this setting. Current system-related factors, such as bed shortages, lead to short LOS and crisis discharges, which likely contribute to a higher rate of readmissions. With the expected ongoing increase in prevalence of schizophrenia spectrum disorders, this study contributes to our understanding of the current patient profile. This will provide a foundation for developing context-specific, tailored strategies to be integrated into the discharge planning processes within the existing healthcare system.
